# Supplementary figures and images for: Biochemical Barriers on the Path to Ocean Anoxia?
Source: mBio. 2021 Jul 13;12(4):e01332-21. doi: 10.1128/mBio.01332-21 (PMC8406191; doi:10.1128/mBio.01332-21)

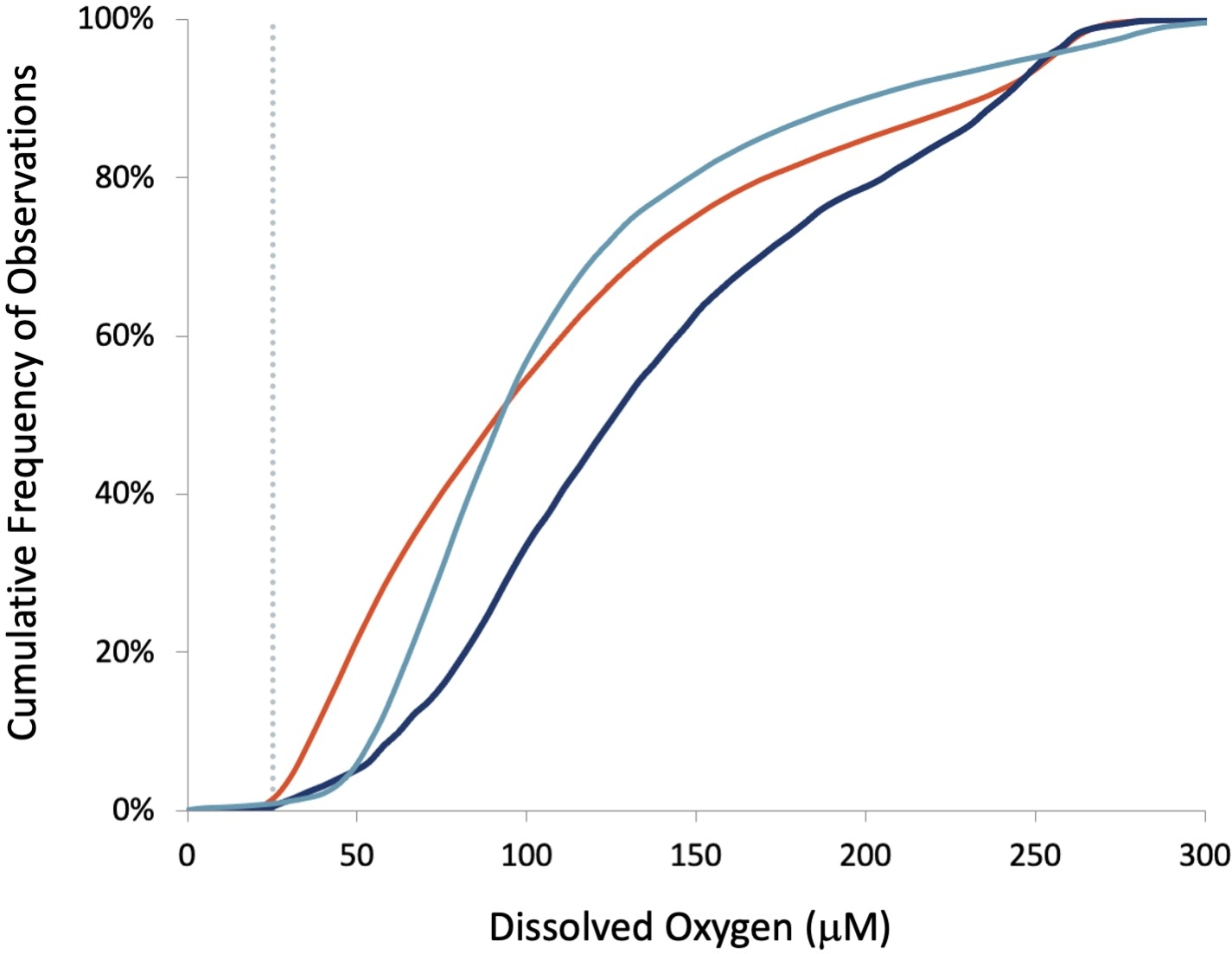

Supplement: FIG S2 [file mbio.01332-21-sf002.tif]

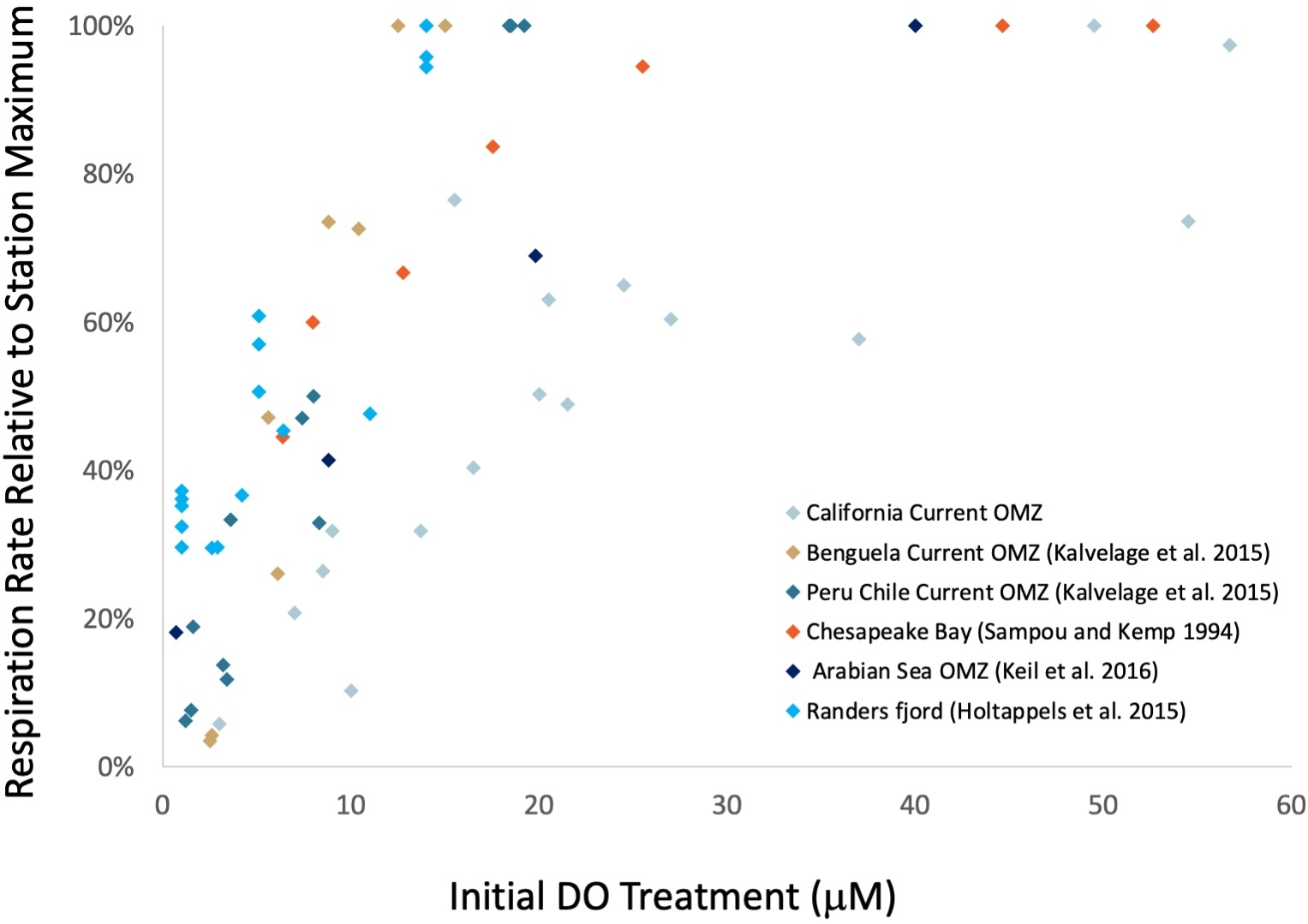

Supplement: FIG S3 [file mbio.01332-21-sf003.tif]

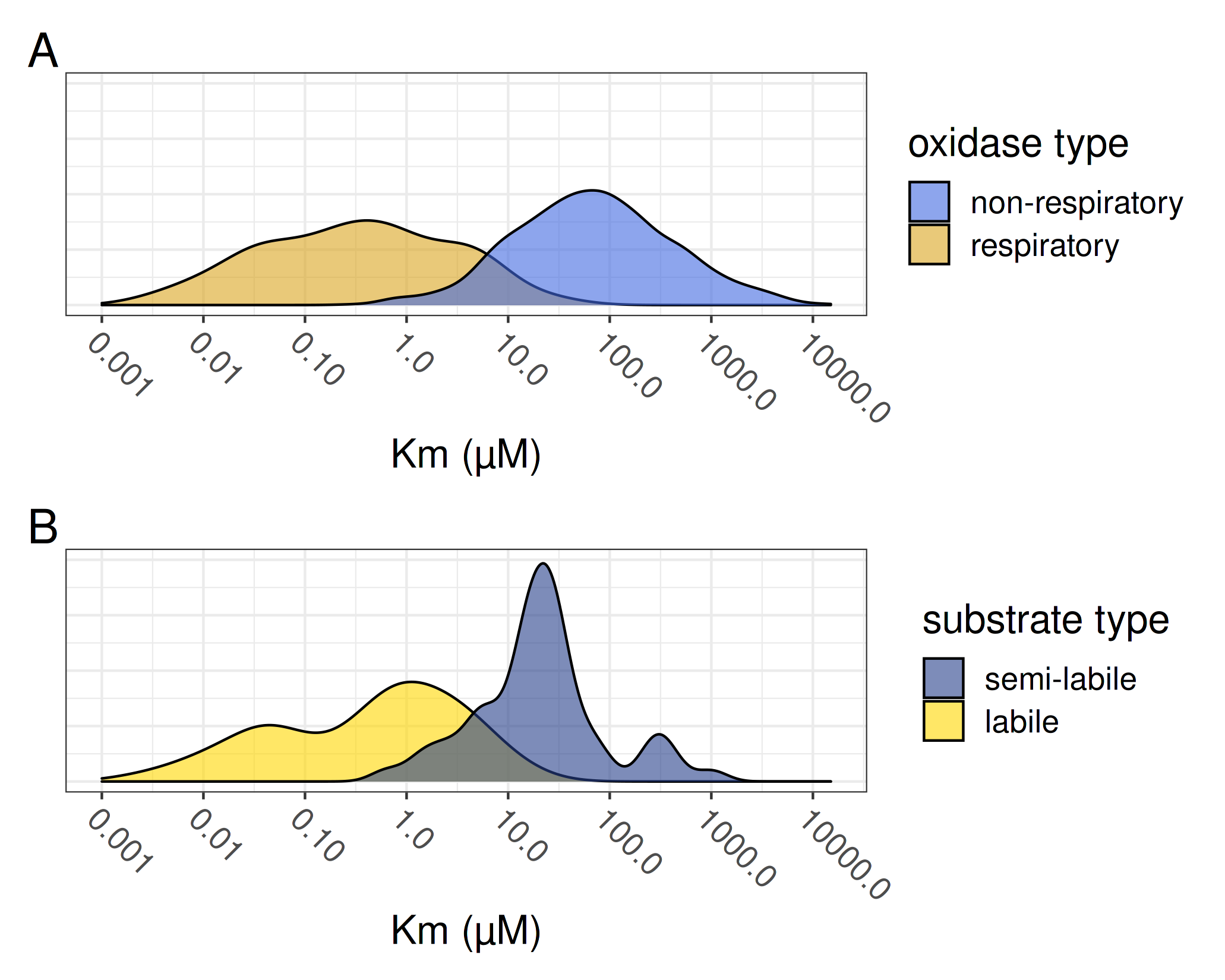

Supplement: FIG S1 [file mbio.01332-21-sf001.tif]
